# The state of art, opportunities and challenges of blockchain in the insurance industry: a systematic literature review

**DOI:** 10.1007/s11301-023-00328-6

**Published:** 2023-02-21

**Authors:** Teresa Dominguez Anguiano, Laura Parte

**Affiliations:** 1 Universidad Nacional de Educación a Distancia (UNED), Madrid, Spain; 2 Department of Business and Accounting, Universidad Nacional de Educación a Distancia (UNED), Madrid, Spain

**Keywords:** Blockchain, Business impact, Insurance, STEEP, Systematic literature review, Technology adoption

## Abstract

Blockchain technologies are quickly changing the competitive business landscape. However, key stakeholders are still sceptical about how, when, and what blockchain might bring to their businesses. This study aims to analyse how blockchain might reshape the insurance industry from an economic and business perspective, as well as to identify which are the challenges and enablers that specifically affect blockchain adoption within this industry, through a Systematic Literature Review (SRL). We also classify existing challenges into five strategic areas: Social, Technological, Environmental, Economic, and Political. Additionally, we provide several recommendations to the manager on identifying the existing hurdles and smoothing transformation.

## Introduction

“In a world of increasing uncertainty and dynamics, the economic and social importance of being insured seems undisputed” (Standaert and Muylle 2022; Stoeckli et al. 2018). The insurance market generated USD 6.3 trillion in 2019 with total global direct premiums growth of 3% and representing 7.2% of the global GDP (Swiss 2020). Despite this positive and solid trend, growing pressure is pushing this market to transform its business for four main factors. The new entrants, such as big tech and insurtechs, with high technology at their core, incorporating new rules on the industry and strengthening the competition; clients, demanding a radically different customer experience; regulators, enforcing capital solvency and legal requirements to ensure customer protection and avoid systemic risks; macroeconomic context, with increasing inflation and uncertain interest rates. The risk landscape is also changing, with a continuously transforming business environment, evolving demographic and social trends, and new technology advancements (Capgemini 2019).

In this context, the insurance traditional industry must embrace this challenging transformation. Emerging technology is considered a key lever to succeeding in business transformation. Among others, blockchain is considered the breakthrough that will support industry disruption (Shetty et al. 2022). An increasing number of studies are focusing on ways of leveraging blockchain to impel change in their business (Zheng et al. 2022). However, academic literature is still scarce and mainly focused on technology (Risius and Spohrer 2017). Besides that, the insurance industry is still underrepresented in blockchain literature (Amponsah et al. 2021).

Our study aims to reduce this gap by exploring how blockchain will impact the insurance industry and by investigating how this technology can address business priorities. The paper examines the current research status of blockchain in the insurance field, by reviewing existing literature, and setting our first objective: to provide an insightful state of the art of blockchain studies in the industry.

Blockchain is considered by some as a game changer (Kar and Navin, 2021) but doubts about that fact also emerge (Hawlitschek et al. 2018). Driven by this uncertainty, our second objective is to offer a comprehensive view of the phenomenon. In doing so, we address the question of how blockchain can potentially transform the insurance business in terms of growth and operational efficiency.

Several hurdles must be jumped to obtain results from blockchain adoption. Even when there are many general blockchain studies, specific detail on insurance impact is limited (Amponsah et al. 2021). To gain a deeper understanding, blockchain challenges and enablers must be adapted to each industry context, as taking general assumptions might throw misleading conclusions, thus wrong strategies. Our third objective is to provide strategic insight into the real impact of barriers and levers to put in place within this industry. Taken together, the study contributes to previous literature in the field and offers several steps to go further.

To answer the research question, the study conducted a SRL by revising documents published in Web of Science, Scopus, and IEEE, from its origins to April 2022. The paper shows how the research is evolving over time, spotting main areas of business growth, operational efficiency, and customer experience, as well as properly weighting the real influence hindrances and identifying enablers in this industry.

The document is organized as follows. Section 2 provides an overview of the blockchain itself and the blockchain in the insurance industry. Section 3 describes the methodology. Section 4 presents the main results. Section 5 concludes the paper and discusses the potential implications and future agenda.

## Background

### Blockchain

A blockchain is a useful tool for business and society. The evolution of blockchain is leading to an attempt to explore the potential of Artificial Intelligence, and the concepts of blockchain-as-a-service (BaaS) by the market leaders for business blockchain solutions (Almeshal and Alhogail 2021). Blockchain 4.0 enhances previous key issues such as latency and scalability (Shrimali and Patel 2022). Their most sophisticated programming versions open the possibility of exploring new business models and reconfiguring the industries, with the following functional and operational features: decentralization, single view of events, immutability, and auditability and transparency.

#### Decentralization

As there is no central organization managing the network or centralizing data storage, the information is shared and governed through the network (Perera et al. 2020). Transaction validation is made by a consensus protocol by all the intervenients of the network, eliminating the need for a third party. The consensus and acceptance rules of the intervenient guarantee trust between participants. Decentralized means that there is no single point where the decision is made. Every node decides its own behavior and the aggregate response of all is the resulting system behavior (Tapscott and Tapscott 2017).

#### Single view of events

The information is synchronized with the network in real-time, providing a single view of events and allowing data to spread across different sites, countries, or organizations (Perera et al. 2020).

#### Immutability

Since each transaction has a link with the previous one (through a hash, cryptographically generated), and transaction validation is performed through the whole network (there is no single point of decision) any attempt to tamper would be easily detected for the other the participants of the network. Participants can add new transactions but cannot modify or delete existing ones (Zheng et al. 2018).

#### Auditability and transparency

Blockchain also confers audibility and transparency because each transaction is recorded with a timestamp. This attribute together with the persistence of the data and the synchronization with the network makes the information of the blockchain easily verifiable and traceable through any point of access to the network (Zheng et al. 2018).

Several categories exist based on access criteria and governance model. Based on the ownership of the network we identify two main categorizations: (i) *Public blockchain* is an open ecosystem where anyone can access, read, modify, or verify the transactions (also called Public Permissionless Network). Most typical examples are cryptocurrencies such as Bitcoin or Ethereum (Clohessy and Acton 2019); (ii) *Private blockchain* relates to one entity or organization that owns the network. It is an ecosystem where only identified and authorized members can alter the network. It has defined governance structures. Private blockchain can be “fully private” when one entity owns the network, or “Consortium blockchain” when it is not a single organization, but a federation, that governs the group (Buterin 2014; Perera et al. 2020).

In between these two definitions, we can find different variants depending on the access allowance. *Public permissioned blockchains*, allow all participants to view the stored data, but only authorized nodes can validate transactions. *Private permissioned blockchain* where read permissions can be restricted to some extent by the owner. Cases such as internal auditing processes or data management or payments reconciliation would lie in this category. Access criteria, governance model, and network ownership shape the network’s technical architecture (Buterin 2014; Zheng et al. 2018), thus its key features. Table 1 summarizes blockchain characteristics depending on the network.

**Table 1:** Blockchain key characteristics

	Public	Private
Access	Permissionless	Permissioned
Consensus	By all the nodes/ miners	By a set of authorized nodes/ entities
Energy Consumption	High	Low
Latency	High	Low
Scalability	Low	High
Governance	Decentralized	Centralized
Decision made	Decentralized	Partly or fully decentralized

Source: Own Please, the setence "Source: Own" at the end of the Table. The setence is outside the Table

### Insurance industry

The insurance business is a crucial contributor to the stability and growth of global wealth. It is one of the essential supports to neutralize costs and aid in case of a disaster, providing a social protection mechanism through financial compensation (Liedtke 2007). Its financial role in reducing the economic uncertainty of society and business allows the population to evolve and take risks. The scale of the industry’s investment, the assets under management or its contribution to the national GDP, and the cited crucial social and economic role it plays, highlights its importance (Liedtke 2007). The COVID-19 has affected many businesses, and the insurance industry is not an exemption. The insurance industry confirmed its market resilience and performed much better than the global economy. In 2020 premiums fell by 1.3%, compared to 5.8% of the global economy. The pandemic increased risk awareness in society, pushing up customer demand and yielding an increase in the importance of this sector in the overall economy. The new normal of hybrid models (physical and digital), has uncovered the difficulties of the insurance business to move online, underscoring the importance of digital transformation of the insurance market.

Despite its overall performance during the pandemic, the industry is facing uncharted challenges, including an emerging loss of trust of the customers, a poor customer experience, slowing growth, and new competition. 

### Blockchain in insurance

The current context is pushing the industry to urgently address these issues by identifying new revenue streams, transforming customer experience, and streamlining operations. The insurance business has traditionally been considered old and slow in innovation (Kar and Navin 2021; Shetty 2022). It has lagged many others in adopting digital technologies to transform its business and embrace customer-focused experience. Blockchain promises to provide unique opportunities to tackle these industry imperatives (Brophy 2019; Grima et al. 2020). Insurance, by its nature, requires a significant effort on back-office tasks. Its operations strongly depend on the interaction between multiple parties (Brophy 2019). The efficiency improvement produced by smart contracts can effectively improve several areas of its value chain.

A smart contract is a contract in which the business logic, e.g. policy conditions in the insurance business, is coded in IT programs that self-execute according to pre-defined requirements (Shrimali and Patel 2022). Blockchain technology also allows developing different organizational forms and new business models, leading to transforming companies’ boundaries blurring industry services (Dal Mas et al. 2020), and allowing envisioning of new service models within ecosystems.

Major insurance companies began evaluating blockchain’s capabilities to support and enhance their core businesses (Grima et al. 2020). Most organizations have gone through user cases without further expanding their adoption, as happens with emerging technologies still not mature. In the last years, we are experiencing the withdrawal of investments of companies who bet in blockchain and are now stepping back (Risius and Spohrer 2017). Many cases died in the Proof-of-Concept state or failed after reaching the real world (Almeshal and Alhogail 2021; Popovic et al. 2020; Shetty et al. 2022). Some examples of real products withdrawn are Guevara, founded in 2013, or Fizzy in 2017 as part of AXA.

Market analysts believe the industry has not yet fully exploited the benefits of this technology (Amponsah et al. 2021). Based on the Gartner hype cycle, there is still a peak of expectations, and cruise velocity has not yet been achieved. There is still a long journey to move to a complete explosion of blockchain applications in insurance (Gatteschi et al. 2018).

With improving blockchain features, the academic community, market experts and practitioners are considering its potential to impact, and be impacted by, the driving forces underlying economic, social, and business developments (Amponsah et al. 2021). Corporations are also shifting their strategy on this technology, from exploration to creating valuable business solutions (Almeshal and Ahhogail 2021).

## Research methodology

This paper provides a comprehensive and cohesive analysis of the status of blockchain in the insurance industry. Our methodology is based on robust frameworks used in previous studies such as Durach et al. (2017), Kitchenham and Charters (2007), Snyder (2019) and Tranfield et al. (2003), including some adaptations.To the best of our knowledge, no previous research in the insurance industry has used a similar approach to synthesize the state of the art, to expose the actual debates in the academy, and to discuss the main results and future avenues. Compared to the traditional literature review, SRL has many advantages, as it reduces bias (Durugbo and Al-Balushi 2022), and ensures transparency and reproducibility (Snyder 2019; Tranfield et al. 2003).

**Fig. 1:**
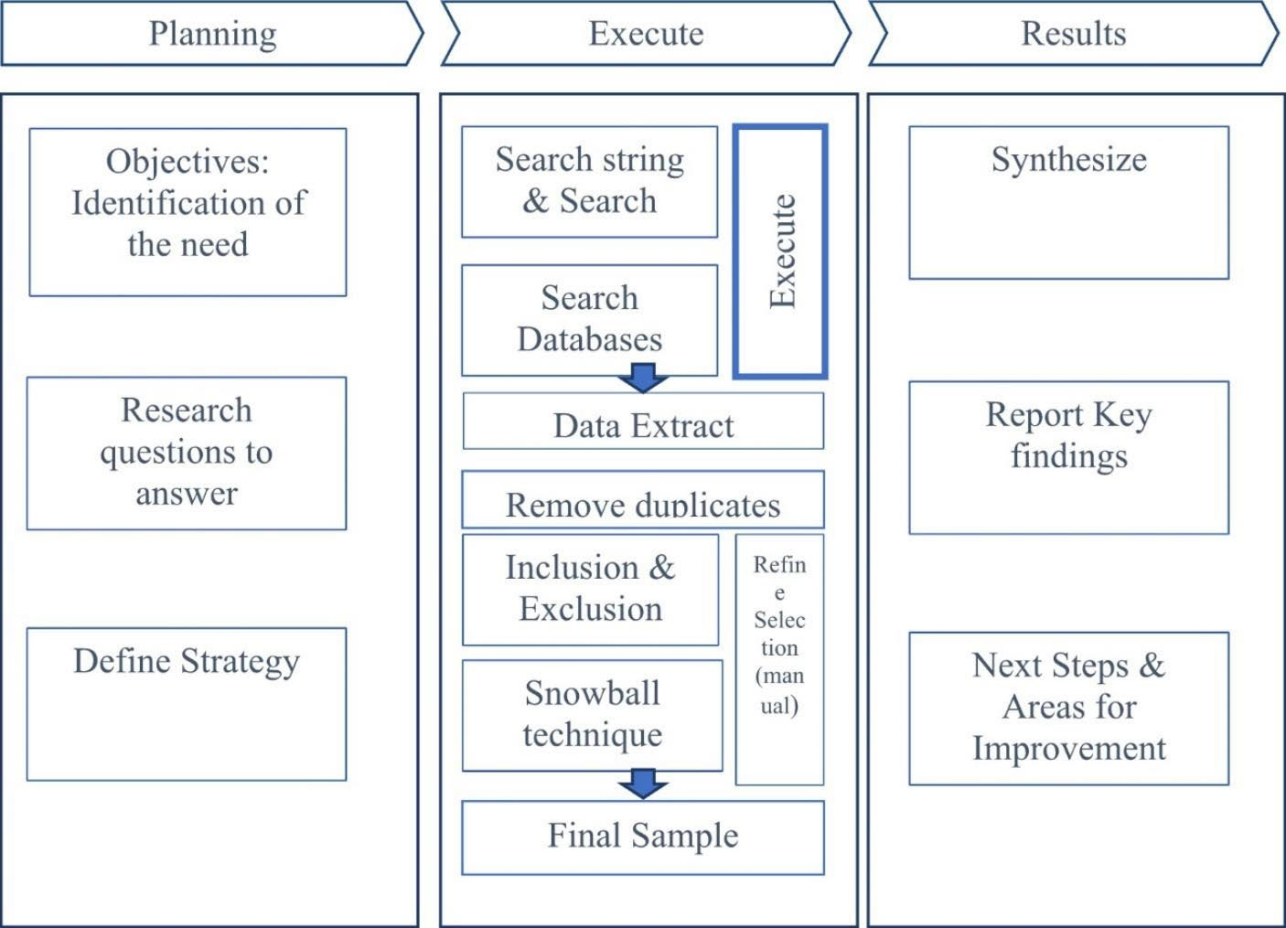
SRL steps. (Source: Adapted from Durach et al. 2017, Kitchenham and Charters 2007 and Tranfield et al. 2003)Please, the setence "Source: Adapted from (....)" at the end of the Figure

In general, systematic literature comprises four main stages: (i) Plan the review, (ii) Identify and evaluate articles, (iii) Extract and synthesize data, and (iv) Disseminate the review results. Drawing on the framework proposed by Durach et al. (2017), Kitchenham and Charters (2007), and Tranfield et al. (2003), our SRL involves the four stages in three main phases with several steps to ensure precise execution of the method: (1) Planning phase, (2) Execution phase, and (3) Results (see Fig. 1).

1. Planning phase

According to Tranfield et al. (2003) the first step is to identify the need to conduct a SRL. An exploratory analysis using databases such as Web of Science, Scopus and Google Scholar confirms that previous academic research in blockchain for insurance is barely existent. Existing research mostly focuses on case studies and prototypes. Indeed, few studies have been conducted in the field of business and management. Therefore, the objective of the current paper is to provide the first systematic literature review on blockchain in the insurance industry. Considering the second step of SRL, the research questions or objectives need to be identified. The purpose of this study is: (1) to provide a comprehensive and cohesive state of art of blockchain adoption in the insurance industry; (2) to offer insights about the application of blockchain in insurance companies; (3) to evaluate the hurdles and enablers of blockchain adoption in the light of real impact in the insurance domain, and (4) to identify future research avenues and challenges.

The third step is to define the strategy for the research. To locate the relevant studies, the search is conducted in Thomson Reuters’ Is Web of Science, Elsevier’s Scopus database, and IEEE. We also include grey literature to provide a more comprehensive picture of the existing research (Casino et al. 2019). Our inclusion criteria comprise articles written in English. We do not select any specific period. Therefore, our article includes all previous papers published in an international peer-reviewed journal related to blockchain in the insurance industry.

2) Execution phase

Firstly, we define the search string and search fields. Due to the nature of the research, the string ends up with very straightforward terms. We chose a tradeoff between larger samples and focus on our area of interest, and after performing several iterations, the *search strings* used to retrieve the data are (“blockchain” OR “block chain” OR “smart contract”) and “insurance” in the *fields* Title, Keyword, and Abstract.

Secondly, we select the Databases according to our objectives. The academic research related to our research interest, business, and management, bases our selection in the databases mentioned before. Once documents are automatically extracted and duplicates removed, we then manually refine the initial sample by applying the exclusion criteria. To do that, we reviewed the Title, Abstract, and entire Paper when needed. Specifically, the exclusion criteria are as follows. *Publication Type*: books, book chapters, conference papers, editorials, or master thesis are excluded; *Industry Focus*: we also eliminate all those articles related to other industries different than insurance, such as health, medicine, or supply chain; *Research Area*: technical papers proposing algorithms, frameworks, or consensus mechanisms that had no interest in business or management are also excluded; *Geo*: to avoid bias based on conclusions from limited geographies, studies have to identify global aspects, and not be locally located.

**Fig. 2:**
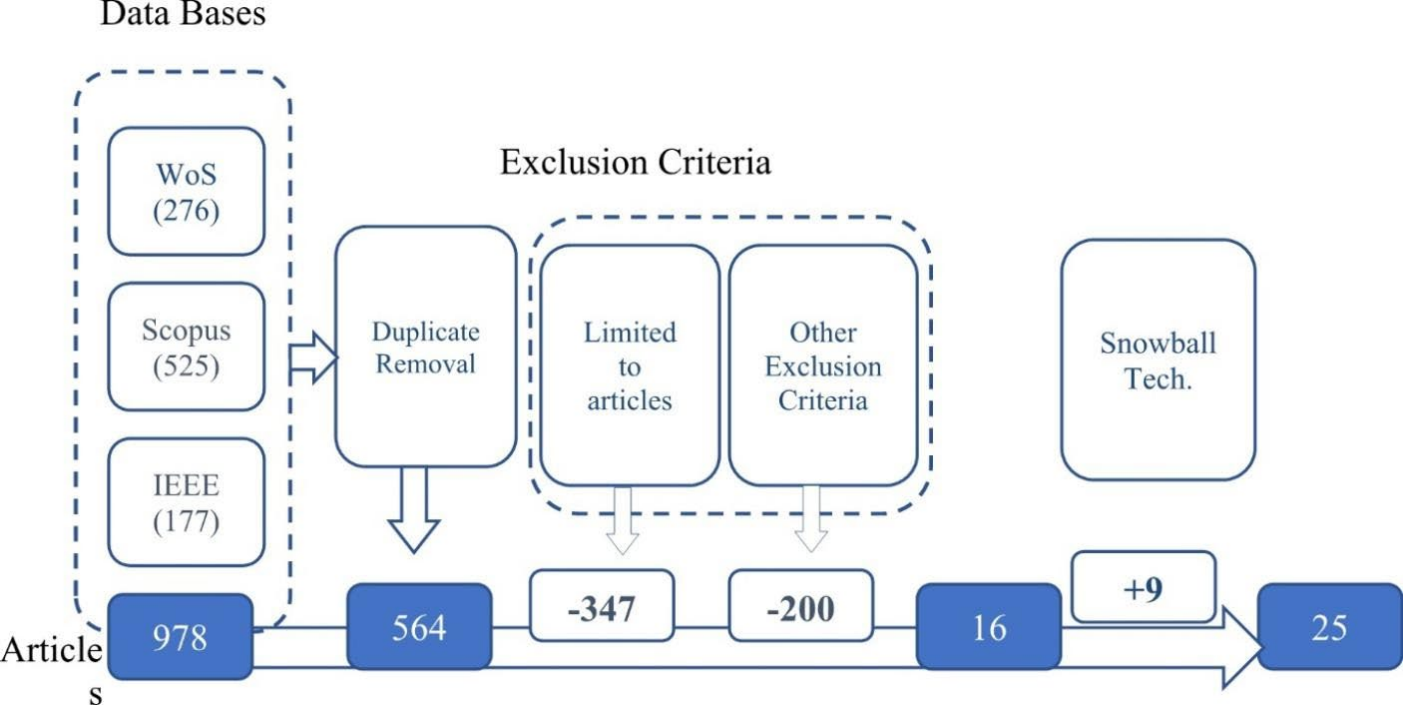
Articles selection process. (Source: Own) Please, the setence "Source: Own" at the end of the Figure

In the execution phase, additional documents are identified through “snowball technique” application (Fig. 2 details all the stages).

The results of the study are presented and discussed in the following section.

## Results

### Research so far

We base our analysis on the remaining sample of the initial search, once publication type exclusion is applied. Table 2 shows the number of articles published per year. It is noted that the first publications in insurance arise in 2017, highlighting a temporal shift between this date and the birth of the technology with Bitcoin in 2009. The highly limited initial programming features of blockchain 1.0, only accurate for Cryptocurrencies and for low application in other domains, explain this lag. With the emergence of smart contracts in 2014 and even more with blockchain 3.0 (Hyperledger) in 2017, scholars and professionals began considering the application of this technology. In 2016, the World Economic Forum and industry leaders first identified the added value blockchain could bring to the insurance industry (Elhence et al. 2022). We also identify a growing interest in the insurance market since initial publications (with a 91% growth in the period 2017–2021). However, blockchain is still underrepresented in academic research in the insurance industry (Amponsah et al. 2021; Risius and Spohrer 2017). Based on the evolution of research published, we expect a race to close this gap in insurance once the whole industry matures, general awareness grows, and pandemic effects cool down.

**Table 2:** Evolution of blockchain publications in the insurance industry. (Source: Own) Please, the setence "Source: Own" at the end of the Table

Publication Year	# Articles
2017	5
2018	18
2019	45
2020	65
2021	67
2022 (*)	17
**Total**	**217**

(*) 2022 includes data to April 22nd, 2022

Another notable finding is the underrepresentation of blockchain studies in areas other than Computer Science and Information Systems (see Table 3). Existing literature is strongly focused on a technical conceptual research, prototypes, and user cases (aligned with Computer Science and Information Systems disciplines), missing other key impacts from a business and society perspective (Risius and Spohrer 2017). This reality, which applies to all businesses, is also evidenced in the insurance industry. Hence, the analysis of the temporal trend by discipline reveals that the focus on technology persists over time.

**Table 3:** Publications in blockchain and insurance by research discipline over time. (Source: Own) Please, the setence "Source: Own" at the end of the Table

Publication Discipline	2017	2018	2019	2020	2021	2022(*)	Total
Computer Science & Information Technology	3	14	30	43	48	7	145
Business & Economics	1	1	9	11	10	5	37
Health Care Science		1	4	6	9	5	25
Government & Law		2	1	4			7
Education	1			1			2
Environment			1				1
**Total**	**5**	**18**	**45**	**65**	**67**	**17**	**217**

(*) 2022 includes data to April 22nd, 2022

Perhaps the technical complexity of blockchain and the lack of academic business frameworks, make publications in nontechnical disciplines very challenging, as experts in this field often lack the technical background. Indeed, little research to date has explained in detail how blockchain works and why this technology can play such a disrupting role in the economy (Risius and Spohrer 2017). We expect growing interest in these areas as blockchain might begin to reshape and define new business models.

Considering the geographic interest, we draw attention to the strong localization of studies in specific countries. Throughout the whole period analysed in this paper, the top 10 countries (23%) report 71% of total studies (see Table 4). Temporal analysis by country also suggests that the interest by country persists over time. Hence, only 8 countries rank in the top 10 for the whole period but also remain during the last year’s rank (Table 4). We also observed China’s increasing attention in the last years when it ranks as the top researcher and generated over one-sixth of total studies (17%). China has also a prominent position in other leading technologies such as Artificial Intelligence, where it also ranks as the global leader (Kaushal et al. 2021).

**Table 4:** Top 10 countries by number of studies. (Source: Own) Please, the setence "Source: Own" at the end of the Table

No. Publications TOP 10 Countries	No. Articles (#Ranking) (2017–2022)	No. Articles (#Ranking) (2020–2022)
INDIA	38 (#1)	20 (#2)
CHINA	31 (#2)	25 (#1)
UNITED STATES	27 (#3)	15 (#3)
RUSSIA	11 (#4)	Did not rank
SOUTH KOREA	9 (#5)	6 (#5)
TAIWAN	8 (#6)	5 (#7)
SAUDI ARABIA	8 (#6)	6 (#5)
ITALY	8 (#6)	7 (#4)
GERMANY	7 (#9)	N/A
UNITED ARAB EMIRATES	6 (#10)	5 (#8)
AUSTRALIA	Did not rank	4 (#9)
SINGAPORE	N/A	4 (#9)
# Top 10	151	97
**% of total publications**	**71% (151 out of 217)Please, one line (do not separate)**	**65% (97 out of 149) Please, one line (do not separate)**

### Key benefits for the insurance industry

We analyze the key benefits of blockchain in the insurance industry by synthesizing results in two main levers for competitiveness: efficiency and revenue growth.

#### Efficiency: operational excellence and cost reduction

Insurance operations comprise many different players. Indeed, the lack of a common interest among a wide number of stakeholders generates bottlenecks. Blockchain technology applied through smart contracts will streamline several functions which are currently spread across numerous systems and databases. The automation of operations will provide a permanent audit trail (Perera et al. 2020). The main difference with existing software is that instead of transferring or copying files between parties, the information is simply distributed in real-time. No single entity owns the information. The whole network owns the information, therefore blockchain functions act as a shared database and serve as a protected, unique source of trusted information. By reducing overlapping activities, eliminating errors, minimizing process leakage, and providing access to a single source of trust, major concerns of the industry are addressed. This back-office efficiency will lead to a reduction in administrative and operational costs (Brophy 2019).  Customer experience will also be strongly enhanced with an increase in trust between the company and customer (Dal Mas et al., 2020), and better service (e.g., see Amponsah et al. 2021; Brophy 2019; Dal Mas et al. 2020; Gatteschi et al. 2018; Grima et al. 2020. According to Porter’s value chain, the cases identified can be classified as primary and support activities.

The primary activities from Porter’s value chain that might be strongly affected by blockchain include claims management, fraud detection, policy administration, and distribution. Considering claims management, the implementation of smart contracts to drive claims processes will lead to the automation of the handling and the pay-out, eliminating the ambiguity of the policy conditions (Amponsah et al. 2021). Lemonade, an insurtech founded in 2015, is a real case of operational excellence applying blockchain. Combining smart contracts and Artificial Intelligence, once a claim is automatically approved, the claimant is paid in seconds (Grima et al. 2020). Another example of how this process has been reshaped is the already-named AXA Fizzy. AXA launched Fizzy in 2017, a parametric product that covered flight delays. By leveraging blockchain techniques through smart contracts and connecting to global air traffic information, the claim process calculated compensations in real time and automatically paid them if policy conditions were met (Kar and Navin 2021 ).

Smart contracts minimize the generation of fraud by implementing the rules of the process, instantly incorporating data proceeding from different external sources (Gatteschi et al. 2018), eliminating the double claiming (Popovic et al. 2020), and providing additional evidence. Fraud is easier to detect as data are immutable and visible (Elhence et al. 2022). Indeed, blockchain improves the faithfulness of financial reporting systems as it is based on shared data from independent entities, a transparent system, and open-access immutable storage (McCallig et al. 2019).

Also, whole policy administration processes can be enhanced (Amponsah et al. 2021), from automatic confirmation of policyholder at the time of issuance, to risk assessment validation from external sources, called oracles, in a smart contract. A successful example is AIG, which associated with Standard Chartered Bank to develop a multinational policy with blockchain as the underlying technology. This reduces friction in a type of business where a lot of manual intervention and multi-country involvement is required (Brophy 2019). 

The ability to disintermediate transactions in the digital world is also crucial to blockchain development. Blockchain nature, and specifically smart contract applications, offers automatic transaction management, thus its adoption will lead to the removal of intermediaries in some specific lines of business (Kar and Navin 2020; Risius and Spohrer 2017).

Regarding support activities, it is fundamental to mention third parties’ reconciliation and auditing. The tasks of data reconciliation between insurance companies and others, such as agents, claims assistance companies, or reinsurers, generate a huge amount of back-office work, producing errors, and implying recurrent validation processes. The implementation of blockchain in these processes will have relevant benefits, eliminating mismatching of data and speeding up the reconciliation process to the desired velocity. The authorized intervenient’s ability to read and write through a private- consortium blockchain where the network validates the data, along with a single view of the data, eliminates the need for double-checking and manual reconciliation. Allianz and Swiss Re who, supported by B3i, have successfully placed the world’s first legally binding reinsurance contract on distributed ledger technology. B3i is an insurance consortium founded in 2016 by key players in the insurance and reinsurance space, to embed blockchain technology into applications for the insurance market. This new product covers one of the core catastrophe reinsurance contracts of Allianz, including key submission parts, final terms, and contractual clauses binding to both parties signing this transaction (Popovic et al. 2020).

Concerning auditing, blockchain is useful for simplifying the job of supervisors. Hence, auditors can benefit from the faithful representation of financial information to assist and provide their opinion (McCallig et al. 2019). Smart contracts can automate regulatory reporting and ensure transparency, consistency, and data quality across carriers. They allow regulators to examine data on new areas of interest or to gain real-time access to signed contracts and the information they contain. 

#### Revenue growth

The insurance industry interacts with many other vertical businesses, such as automotive, logistics, health, and finance, and owns a huge amount of data. This industry is in a unique position to take advantage of its role in the ecosystem and use the huge amount of data it possesses (Njegomir et al. 2021). This could transform the present interaction with end users, limited to policy issuance, renewal, and claims management, into a richer experience. The combination of blockchain and different trending technologies such as Artificial Intelligence, Cloud Storage, and Internet of Things (IoT) among others, yields an uncharted world for business and product innovation (Amponsah et al. 2021; Shrimali and Patel 2022), with the ability to create new business models , and a new approach for markets and customers (Gatteschi et al. 2018). We explain the most relevant cases for the insurance industry in the following paragraphs:

*Peer- to – Peer Insurance*, this modality goes back in time to the concept of risk pooling, contributing to “self-policing” between the insured. In this model, a group of individuals pools their premiums to jointly get insured against a risk (Brophy 2019). Blockchain provides the trust needed between the intervenient without a third party acting as a validation. As claims are approved by a majority of the network (Amponsah et al. 2021), this model ensures a more diligent behavior by sharing this responsibility, eluding fraud generation. A case of peer-to-peer insurance successfully launched is Friendsurance (Amponsah et al. 2021; Brophy 2019). In 2010 they pioneered a Peer-to-Peer insurance model which rewards individuals for being claims-free (https://www.friendsurance.com/). Based on existing examples and its evolution there is still a long way to go for this business model to expand.

*Microinsurance*, cost reduction by blockchain gives a new chance to reach the underserved, not only to underinsured countries but also to the marginal society in the first world (Dal Mas et al. 2020). Blockchain technology, with its more reliable and inalterable alternatives to current registries, can allow access to new markets in regions with a lack of good data and high grades of corruption. The dramatic reduction in policy lifecycle costs also provides a platform for new low-cost products “on demand” such as bike or scooter rental for ride insurance (Dal Mas et al. 2020).

*New products and new pricing calculations* will slowly replace existing rating engines for many products. The most traditional engines base results on demographic factors, but in the last years, the commercialization of products based on usage (UBI) without the need for blockchain technology, has expanded. Blockchain will allow sharing of all information between different carriers, such as car manufacturers, weather forecasters, and insurance with the trust of the network.

*Ecosystem*, the dilution of industry boundaries due to the exponential growth of data and new technologies provides opportunities in the insurance market to generate new revenue streams out of their core services. Future business models based on blockchain will particularly enable new services out of the traditional scope of vertical industries (Risius 2017). An example of an End-to-End Network can be found in a Marine Insurance Policy. This is a complex business, with a worldwide scope and many different stakeholders including logistics companies, port authorities, multicountry legislators, insurers, reinsurers, and customers, all taking advantage of these cutting-edge technologies. The logistic giant Maerks has partnered with insurance companies MS Amling and XL Catlin Willis- Tower Watson as insurance advisor, Ernst & Young as consultancy firm, and Guardtime as software provider, to create a maritime insurance platform blockchain, that helps managing the risk and the shipping (Brophy 2019).

### Challenges of the industry to adopt blockchain

The potential impact of blockchain goes far beyond technology, playing a role in the political, humanitarian, social, and scientific landscape (Swan 2015). The introduction of blockchain, as with any other innovative technology, is not without risks. It faces multiple challenges in several areas, such as regulation, social, business, and technical (Werner et al. 2021). Based on the impact broadness of this technology, we identify the STEEP framework as the most accurate one to map adoption´s hurdles and enablers. We previously discarded other frameworks as they did not fully support the research objective.

The STEEP analysis provides an appropriate level of analysis with the ability to easily foresee the factors. This framework has been extensively used in marketing and strategy management. Grima et al. (2020) successfully applied STEEP to analyze the factors impacting blockchain adoption in the insurance industry. This framework helps in obtaining a comprehensive view of the influences and existing or future threats. By identifying those threads and any shortcomings, the insurance industry will be better prepared to respond and appropriately manage existing challenges.

As part of this work and following the previously mentioned study from Grima et al. (2020), we have adapted the framework to include internal aspects that influence blockchain adoption under environmental factors. Based on the paper published by Ho (2014), and including the Grima et al. (2020) adaptation, we provide below comprehensive details of the social, technological, economic, environmental, and political factors.

#### Social

Social factors include the social, cultural, and demographic aspects of the external environment, such as gender, age, and educational level, including its impact on consumer attitudes and willingness to change. Even when social factors play a key role in innovation technology adoption, its impact on the pace of blockchain in the insurance industry, at least at the first stage, is limited. The initial expansion of blockchain will be driven by cost efficiency, and therefore will streamline insurance operations without radically disrupting existing processes (Kar and Navin 2020). While this factor is less crucial in this sector than in others, a positive public perception of blockchain will benefit all industries.

Several scholarships have analyzed society’s willingness to adopt new technology. Status quo bias is considered the most relevant factor. This bias is driven by three key factors: The first factor is the *uncertainty costs*, as a perception of the risk of blockchain adoption. The lack of knowledge of this technology yields doubts and concerns when embracing it. For example, fear of security breaches or uncharted risks (Grima et al. 2020); the second factor is *loss aversion*, whose theory explains that loss aversion can affect human decision-making. Consequently, individuals can take decisions trying to avoid losses, and blockchain adoption can be considered risky, as data sharing might be a concern for the network participants (Wang et al. 2023); finally, the third factor is *physiological commitment*, where social proof theory states that users act relying on the experience of others. Like other innovations, once the number of users increases, their network converts into a more prone audience, generating a snowball effect (Dal Mas et al. 2020). Blockchain awareness keeps general interest and becomes better known and popular. Hence, the exchange of digital assets as an irrupting business (Risius and Spohrer 2017) with NFT Metaverse, impacting media and expert audiences such as investors and technology advisors, accelerates the general awareness of this technology.

AXA Fizzy is an example of a launch of a blockchain product visible to customers with an unsuccessful end. According to the managers of the initiative, the product was closed after two years life because there was not enough market appetite (Seneviratne et al. 2020), showing the difficulties to introduce disruptive technology in society.

To reduce status quo bias and generate a positive impact on customers’ decisions when adopting blockchain, the insurance industry will need to put in place an implementation that ensures success. Special attention to communicating and guaranteeing security and data privacy will be essential to attract sufficient users to create a positive network effect.

#### Technological

Technology encompasses several factors, such as the maturity of the technology, infrastructures, and technological changes, that affect the internal and external environment. Like all emerging technologies, blockchain suffers several design and architectural limitations, which inhibit its widespread adoption and are a showstopper for specific cases. These restrictions’ weight differs from one industry to the others. Academics identify eight limiting factors in the design and maturity of blockchain technology: *Efficiency*, in terms of speed of processing data and transactions (Almeshal and Alhogail 2021); *Energy consumption*, miners demand high power processing time to find and apply cryptography (to mine) in each of the blocks in a public blockchain. As the blockchain becomes larger in size, there is a need for more processing capabilities to mine the blocks; *Scalability*, as the number of transactions increases blockchain systems must scale efficiently to achieve widespread adoption within financial services (Perera et al. 2020; Zheng et al. 2018).

*Storage*, blockchain must go hand in hand with cloud storage to effectively maintain and process such a huge amount of data. Deciding to move to the cloud at the early steps of a new IT system is a quite straightforward action. Most insurance companies have already an IT system for decades. In this context, the strategy to move to the cloud has its difficulties and needs to be carefully analyzed (Grima et al. 2020). To maximize efficiency and scalability and reduce energy consumption and storage needs, companies are opting for private blockchain. They can take advantage of lighter consensus mechanisms as the stakeholders of the network are previously validated and participants are already trusted, allowing higher scalability and more speed and efficiency, without jeopardizing security (Risius 2017); *Security*, the possibility of blockchain being hacked by third parties generates concern in the -industry for specific cases (Walsh et al. 2021). Besides Peer-To-Peer insurance and IoT combined with blockchain, smart contracts depending on external oracles is another outstanding case on which data security plays an essential role.

*Technology standardization*, as technology is quite immature (Radanovic and Likic, 2018), there is a lack of an industry-wide definition with no clear winning solution (Malhotra et al. 2022; Walsh et al. 2021). It is unlikely that a single blockchain system will operate globally (Shetty et al. 2022; Treiblmaier et al. 2021) and executives’ fear of a wrong selection is preventing action, as opting for an erroneous technology would have a tremendous negative impact; *Interoperability*, with the existence of a multitude of different protocols (Sakho et al. 2019) the coexistence between different blockchains is still an unresolved issue. The success of the interoperability between blockchain platforms will play a key role in the pace of adoption (Risius and Spohrer 2017). Issues regarding interoperability and standardization are a matter of maturity evolution and global adoption, led by technology providers.

*Integration with legacy systems*, this challenge strongly affects the insurance business, where traditional companies normally hoard countless complex legacy systems. Legal requirements and operational needs impose record-keeping standards that make it impossible to shut down the IT systems that keep the policies’ historic records. For the same reason, the very common M&A operations in the sector usually inherit acquired core business applications. Both factors determine a very complex IT landscape. Integrating new technology with existing IT processes and legacy systems is one of the most challenging and costly topics to be managed (Gatteschi et al. 2018; Shetty et al. 2022), with high switching costs, in terms of people, economics, and time. This concern has been barely investigated by researchers (Risius 2017).

#### Economic

We identify three global trends that might impact blockchain adoption within the insurance sector: Market liquidity, New business models, and Environmental, Social and Governance (ESG) inclusivity insurance.

The COVID-19 has changed market liquidity. It has also accelerated the digitalization transformation among companies. In this context, the appetite for investing in new technology, including blockchain, is speeding up the resolution of technical issues, and creating new solutions. Global digitalization is also generating new business models (Riasanow et al. 2021) with an impact on the economic environment. In this sense, blockchain can dramatically accelerate the creation of transversal ecosystems, where companies interact across the value chain blurring industry services boundaries and establishing new relationships between organizations (Riasanow et al. 2021). Also, intermediary services in Multisided Markets such as gathering information, negotiating, and managing relationships could be fully provided by blockchain implementation through complex smart contracts (Risius and Spohrer 2017). Finally, ESG inclusivity insurance is a global demand and mission for the industry. Habits traceability (IoT with blockchain) will ensure appropriate conduct, and cost reductions on policy management from operations streamlining will yield more affordable premiums. Therefore, part of the currently excluded society could be incorporated into insurance, providing a global benefit (Dal Mas et al. 2020).

#### Environmental

Environmental aspects involve internal industry status, organizational culture, and readiness of the sector. It considers both intra-organizational and “ecosystem” implications. We highlight the main aspects: *Environmental impact*, energy consumption generates concerns about the usage of this technology, but this fact only affects to public networks such as bitcoin (Dal Mas et al. 2020); *Cost*, considering Transaction Cost Theory (TCT), blockchain technology is an enabler for reducing overall costs because they decrease the cost of transactions (Dal Mas et al. 2020); *Intra-organizational factors* are considered the most relevant factor for business transformation. Several aspects such as **s**trategy, change management in culture, and organization, are outstanding pillars for business transformation.

In terms of strategy, many insurers have a limited understanding of blockchain and do not visualize how it will boost their business growth strategically, missing a long-term approach to blockchain adoption (Risius and Spohrer 2017; Shetty et al. 2022). Insurance companies need to establish a roadmap for blockchain implementation aligned with their business strategies. Blockchain prototyping can be a part of the pre-adoption phase, supporting business decision of whether embrace the technology or adopt a “wait and see” position (Ostern et al. 2022). It is key to identify quick win strategies, which will provide investments within the company and facilitate internal selling, together with a long-term vision.

Concerning change management, blockchain-based systems and their overall implications demand a change in thinking and in the culture and organization of traditional carriers (Walsh et al. 2021). One of the levers for this transformation is communication and sponsorship from the top management. Currently, executives face difficulties in finding and communicating the benefits of blockchain for three main reasons: (i) difficulties to evaluate and confirm a positive revenue stream as there is not a clear link between this technology and the economical business benefits (Risius and Spohrer 2017; Schweikl and Obermaier 2020); (ii) lack of real cases, with an absence of a clear “buy-in” (Risius and Spohrer 2017); (iii) difficulties in understanding blockchain, making insurance employees not feeling comfortable when explaining to a colleague (Popovic et al. 2020).

Regarding organization, insurance companies are traditionally organized in vertical siloes aligned to a specific line of business, and with cross structures limited to support activities. This complicates the introduction of such an innovation as blockchain. Incumbents experience Status Quo Bias when it comes to the adoption of blockchain solutions (Walsh et al. 2021). Employees, based on their top management priorities, are strongly focused on immediate vs. delayed results and technological uncertainty is preventing people to go for the change. The lack of expertise and talent with difficulties to engage skilled resources in such a novel and complex technology (Amponsah et al. 2021; Popovic et al. 2020), is another stopper for progress. The fear of being displaced also generates internal resistance (Malhotra et al. 2022). Organizations must provide resources (in terms of people and budget allocation) and train employees to get up to speed, understand the technology (Walsh et al. 2021) and reduce the Status Quo Bias (Godefroid et al. 2022). Currently, a lack of support within the organizations is detected (Popovic et al. 2020). Top managers will need to sponsor and commit to blockchain-based systems (Walsh et al. 2021), playing a change’s agent role to overcome internal resistance (Godefroid et al. 2022).

*Ecosystem readiness*: As insurance deals with many stakeholders interacting with several industries, ensuring the readiness of the whole value chain intervenient will be key to adopting blockchain. Even when the automation and control of blockchain have the potential to enhance cross-partnership (Akyuz and Gursoy 2020), there is evidence of the lack of involvement of key roles in the industry (Brophy 2019). We highlight the importance of four intervenient: intermediaries and loss adjusters, reinsurance companies, partners, and oracles. *Intermediaries and loss adjusters* stay behind on the transformation. There might be a fear of disappearance or simply a total lack of skills and knowledge. *Reinsurance companies* are leading the change in the traditional stakeholders, being the most prepared to adopt the change. Working with *partners* in a novel technology is with lots of uncertainties. Risusis and Spoher (2017) point out the difficulties in setting the power in industry-wide blockchain systems. The governance model, each player’s role, and ensuring that every participant benefits from the ecosystem will be crucial to exploring new partnerships. *Oracles* are essential for smart contracts as external data must be injected into the blockchain. The key challenge with oracles will be the need to have a strong reputational system and governance mechanism.

#### Political

According to insurance executives, grey zones in regulation are one of the key stoppers for blockchain adoption, as companies fear entering this new technology (Walport 2016). In most countries smart contracts do not currently have legal validity, preventing the expansion of this forward technology (Gatteschi et al. 2018). The legal recognition of blockchain-based private arbitration as a binding decision will be a cornerstone in the evolution of the industry (Treiblmaier et al. 2021), as smart contracts are one of the most powerful applications of blockchain in insurance. Another concern is the aim of some governments of extending existing laws and legal constraints to a new concept of business. There is a discussion around the real possibility and even the desirability of such a law transposition (Risius and Spohrer 2017). Also, the inexistence of cross-country laws difficult the use of blockchain for cross-border operations (Amponsah et al. 2021; Malhotra et al. 2022), as it is difficult to settle in which country a smart contract is legally binding (Shetty et al. 2022). While legislation is not yet in place, sandboxes as a workaround, are spreading throughout different geos. Sandboxes are a secure environment to test new businesses, speeding up the process of regulating and allowing a “test & learn” approach (Brophy 2019). Policymakers will need to balance between providing avenues for new technology and minimizing systemic risks for blockchain adoption (Yeoh 2017). Regulation will be a showstopper or a key enabler depending on how governments control this technology.

In terms of insurance, worldwide Insurance Institutions are generating awareness and helping with new legislation. For instance, EIOPA (European Insurance and Occupational Pensions Authority), as the European Union Financial Regulatory Institution for Insurance, is making attempts to provide a clear definition of Peer-To-Peer insurance for legal purposes and generate awareness on the use of blockchain to facilitate the new regulation (Brophy 2019).

## Conclusion

Our study provides a holistic view of blockchain adoption in the insurance industry. It contributes to a better understanding of the challenges and enablers that specifically affect this sector. The study also offers a research agenda based on scientific evidence and several guidelines for insurance executives to set their blockchain strategy. To the best of our knowledge, this is the most comprehensive study of the current literature in the insurance industry, through a systematic literature review, with a cross-discipline assessment.

We conclude that, despite the uncertainty, blockchain will be a game changer for the insurance industry. Blockchain’s “foundational” features (decentralization, single view of events, and immutability) and the unique position of the insurance business (managing relevant amounts of data and interacting with many other sectors), can extend insurance carriers’ traditional services to new avenues. The adoption of blockchain and the unique value proposition might allow insurance carriers to become the network’s orchestrator for new cross-industry ecosystems, be a solution for insuring the uninsured through microinsurance, or generate innovative products based on new rating models and claims management.

Blockchain can also enhance internal operations, such as auditing, reconciliation processes, claims management, or policy administration. It has the potential to streamline core operations, eliminate errors, and minimize paperwork for all stakeholders, leading to a reduction in overall costs as well as an enhancement in customer satisfaction.

Existing cases of blockchain in the insurance business have mainly stopped at a “Proof-of-Concept” state or have been discontinued in production. The current state and hurdles identified show an unclear timeframe for blockchain extensive adoption. There is still a long journey to evolve from the current situation to a tangible business transformation.

This transformation is not without risks. This study points out a set of social, technological, economic, environmental, and political challenges to be managed before this technology becomes disruptive. Our results emphasize the importance of technological and organizational hurdles lowering social impact in this industry. Companies will need to overcome general technological barriers associated with any innovative technology, as well as make a shift in their operations, organization, and culture.

This study predicts a continuity in the line of adoption, focused on internal operations with cost efficiency as the main driver of the decision. Consortium blockchains also seem to be a prominent area for progress, while new business models, such as peer-to-peer insurance or new ecosystems, will be challenging in the short term because of their impact on social behaviours, the large number of stakeholders involved, and the more complex technical requirements.

Insurance carriers will need to decide their pace on blockchain adoption. To set up an insightful roadmap, they will need to carefully identify, if, and how, blockchain will support their strategic plan. It is mandatory to align this vision with their ability to execute, based on their current capabilities. This transformation will need to balance quick wins with long-term milestones, ensuring resources are in place for all the workstreams, and consider the maturity of this technology and their organization.

Like any other study, our paper has some limitations. We investigate the challenges and the expected value of blockchain adoption in the insurance industry based on a systematic literature review. While the study contributes to explaining the adoption of blockchain from a theoretical perspective, it lacks empirical analysis.

As in any other science, it is crucial to support the theory with experimentation. Only with the complete picture, we will be able to provide the most appropriate solutions. Therefore, it is worth mentioning the need to conduct case studies and other empirical investigations as a future research direction. This could include analysing success and failure cases, how carriers are setting the strategy and the pace of implementation, how they are implementing it, and which challenges they are facing. This could help provide personalized guidelines to companies based on their specific context (size, line of business, geos, etc.).

As mentioned in the study, we also recommend future researchers to focus on a deeper understanding of organizations’ readiness and approach to blockchain adoption. Organizational and cultural challenges are considered crucial to a successful transformation. Incorporating change management and business behavior methodologies will bring light to this process, and will reveal findings that strongly help insurance carriers in their transformation roadmap.

Another avenue for future studies is defining a conclusive framework for measuring this technology’s added value. Tackling the initial investment costs and finding a way to measure those more intangible benefits, such as ease of use or trustfulness, will help carriers with their decision and follow-up processes.

Furthermore, the potential of blockchain technology to contribute to environmentally sustainable development goals (SDGs) is an area of particular interest not only for insurance companies but also for the society. It might be interesting to extend the handful of existing studies on this particular topic (such as supply chains, smart cities, and specific SDGs) to offer an insightful view of this global challenge.

Lastly, we invite future researchers to replicate this framework in other industries with little evidence to date. Although blockchain is considered one of the most remarkable innovations in the 21st century, it is still necessary to have a complete understanding of the benefits for all economic activities, industries, and markets.

## Data Availability

Not applicable.
